# Using Neural Circuit Interrogation in Rodents to Unravel Human Speech Decoding

**DOI:** 10.3389/fncir.2020.00002

**Published:** 2020-01-30

**Authors:** Demetrios Neophytou, Hysell V. Oviedo

**Affiliations:** ^1^Biology Department, The City College of New York, New York, NY, United States; ^2^CUNY Graduate Center, New York, NY, United States

**Keywords:** speech-brain, auditory cortex (AC), animal models-rodent, cortical circuit, temporal processing and spectral processing

## Abstract

The neural circuits responsible for social communication are among the least understood in the brain. Human studies have made great progress in advancing our understanding of the global computations required for processing speech, and animal models offer the opportunity to discover evolutionarily conserved mechanisms for decoding these signals. In this review article, we describe some of the most well-established speech decoding computations from human studies and describe animal research designed to reveal potential circuit mechanisms underlying these processes. Human and animal brains must perform the challenging tasks of rapidly recognizing, categorizing, and assigning communicative importance to sounds in a noisy environment. The instructions to these functions are found in the precise connections neurons make with one another. Therefore, identifying circuit-motifs in the auditory cortices and linking them to communicative functions is pivotal. We review recent advances in human recordings that have revealed the most basic unit of speech decoded by neurons is a phoneme, and consider circuit-mapping studies in rodents that have shown potential connectivity schemes to achieve this. Finally, we discuss other potentially important processing features in humans like lateralization, sensitivity to fine temporal features, and hierarchical processing. The goal is for animal studies to investigate neurophysiological and anatomical pathways responsible for establishing behavioral phenotypes that are shared between humans and animals. This can be accomplished by establishing cell types, connectivity patterns, genetic pathways and critical periods that are relevant in the development and function of social communication.

## Introduction

During the Yugoslav wars of the 1990s ordering coffee could get one killed. In a Croatian coffee shop, the “*Kava*” pronunciation was associated with being a Croatian Catholic and led to the purchase of coffee without incident. But the “*Kahva*” pronunciation was associated with Bosnian Muslims and could potentially result in a bullet to the head (Dragojevic et al., 2015). This is not an isolated example: from Ebonics to the interpretation of the Constitution of the United States, language is of paramount importance to humans. Whether or not one believes that language fundamentally constrains how an individual conceptualizes the world (Boroditsky, 2011), it is clear that it at least requires developmental programs that wire specific neural circuits. In this review article, we will briefly explore some widely-recognized computations the human brain performs to decode social communication and describe the animal studies designed to dissect potential circuit mechanisms that could serve as a blueprint to understand these processes.

Systems in the brain undergo time-limited periods of postnatal plasticity where synaptic connectivity adapts to the demands of the organism’s environment. For instance, in the rat primary Auditory Cortex (A1), frequency representation (tonotopy) develops shortly after hearing onset in a time window between postnatal days 9–28. Rearing pups in white noise during this period can irreparably degrade spectral tuning (Zhang et al., 2002). Similarly, unnatural auditory experiences due to disease in early human infancy lead to degraded language abilities (Centers for Disease and Prevention, 2010). The implication of these findings is that exposure to the statistical structure of social calls provides the training signals that guide connectivity (Rauschecker, 1999; Levy et al., 2019). During critical periods, the brain relies on the statistical regularities of the auditory world (some sound sequences are more probable than others) to shape neural circuits in order to make detection and decoding of ethologically relevant signals faster in the future. In this review article, we focus mainly on studies from A1 (unless stated otherwise) because it is the first auditory area believed to represent perceptual features of sounds directly involved in decoding social communication (Wang et al., 1995; Nelken, 2008).

## Circuit Foundation of Phoneme Detection

There is general agreement regarding the basic computations a brain must perform to decode social communication. Mechanisms must exist to rapidly decode and bind phonetic elements and their temporal boundaries in a sequential and hierarchical manner. Studies in humans have made great advancements and fundamentally shape how we think about language processing (Yi et al., 2019). For decades it was unclear what was the most basic unit of speech that is decoded by neurons in A1. Recent studies using human cortical surface recordings (ECoG) from the superior temporal gyrus (STG) have discovered selectivity to phonemes in neural responses. Phonemes are the most basic units of speech sounds that have semantic meaning, and in the human STG responses are systematically organized by phonetic feature category (Mesgarani et al., 2014). Achieving this precise spectrotemporal tuning would require the wiring of phoneme detectors: neurons that preferentially respond to specific spectrotemporal features.

**Figure 1:**
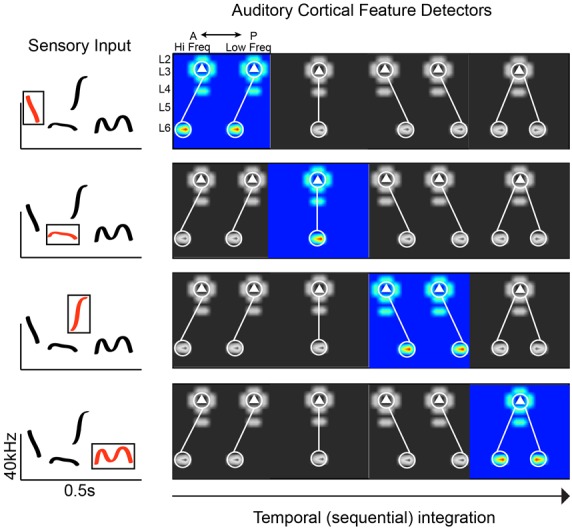
Plausible connectivity motifs of phoneme detectors. The ACx must extract revelant spectrotemporal features from sensory input. In this hypothetical example, we show a spectrogram with common mouse vocalization motifs (left panel). On the right are examples of connectivity motifs mapped in L3 of the mouse ACx. Each highlighted motif would be preferentially activated by a particular feature (“phoneme”) in mouse vocalization (red traces in boxed region, left panel).

Given the limitations of experimental work in humans, we can more thoroughly interrogate potential circuit mechanisms in animal models that utilize social calls (e.g., birds and rodents). Circuit-mapping studies in the rodent A1 have begun to reveal connectivity schemes consistent with phoneme detection (Oviedo et al., 2010; Levy et al., 2019). Social calls are spectrotemporally complex, requiring neurons to integrate across frequency channels. The superficial layers in A1 have been postulated to be a hub of spectral integration (Metherate et al., 2005; Winkowski and Kanold, 2013). Using circuit-mapping *in vitro*, we discovered along the tonotopic axis of the mouse A1 that Layer 3 (L3) excitatory neurons preferentially receive out-of-column inputs from neurons in L6 at higher frequency bands. The distance of this asymmetric L6 pathway ranges from 200 to 400 microns. Given the size of the mouse A1 (just over 1 mm along the horizontal plane covering 6 octaves; Guo et al., 2012), the L3 cells receiving spectrally shifted input from L6 neurons could be integrating across 1–3 octaves. This asymmetric pathway is absent along the isofrequency axis (orthogonal to tonotopy), where we found inputs that are columnarly organized. Therefore, the functional anisotropy of A1 is directly reflected in the connectivity of neural circuits. We also found that differences in circuit-motifs correlate with differences in responsiveness to simple stimuli: L2 cells (which receive columnar input) show well-defined frequency tuning to pure tones, whereas L3 cells are largely unresponsive to pure tones (Oviedo et al., 2010). Though on average there are consistent circuit-motifs in A1, the correlation of input maps between pairs of neighboring neurons is very weak (0.3 within 100 microns, compared to 0.7 in the barrel cortex; Shepherd and Svoboda, 2005). Neighboring neurons can also respond very differently to the same stimulus (Hromádka et al., 2008; Oviedo et al., 2010). This could translate into a greater diversity of spectrotemporal decoders that maintain response flexibility (Figure 1). A relevant question is whether these circuit-motifs are unique to A1, or are also found elsewhere in the cortex. Compared to other cortical areas that have been mapped in detail, it does appear that A1 has a number of unique motifs (e.g., the aforementioned asymmetries). But the broader observation is that some circuit features are conserved across cortical areas (e.g., recurrent connections and a spectrum of columnar information flow), and some are unique to facilitate specific computational demands (Dantzker and Callaway, 2000; Bureau et al., 2006; Weiler et al., 2008).

## Representation of the Multiscale Temporal Features of Speech

Social communication contains information at multiple time-scales, from fast fluctuations used for sound localization (~1 kHz), to slower modulations that correlate with word and syllabic structure (1–30 Hz). Different populations of cortical auditory neurons have shown distinct preferences to spectrotemporal modulation rates. There are neurons that represent amplitude modulations through the synchronized timing of their spikes (Wang, 2007; Wang et al., 2008). This synchrony is observed in response to slow temporal modulations (<5 Hz), which are associated with word and syllable boundaries (Arnal et al., 2015). Other populations represent amplitude modulations through a mean population firing rate. This rate code is observed in response to fast temporal modulations (<30 Hz) associated with phonemes (Arnal et al., 2015).

Moreover, the brain needs to rapidly decode multiplexed speech information before it is overwritten by new input (Christiansen and Chater, 2016). This would ensure that we are able to comprehend the entirety of the speech signal and keep that information in a temporary buffer. One potential strategy is to recode the sensory input as it comes in, to capture each important component of the signal before a new input interferes (Brown et al., 2007). This results in a compressed representation of the information and minimizes the impact on echoic memory (sensory memory specific to audition; Pani, 2000). A candidate neural mechanism to accomplish this compression occurs in the form of synaptic depression at the thalamocortical synapse, where neural activity is transformed from explicitly representing the temporal structure of sound (i.e., subcortical input) to a rate code in A1 (Gao and Wehr, 2015).

To minimize the effects of echoic memory constrains aforementioned, the auditory system chunks the information into increasing levels of abstract representations of the sound during perception (Christiansen and Chater, 2016). It has been proposed that this could be achieved with increasing temporal windows along a hierarchy to allow for an accurate representation of the chunks (see the section below for details; Hasson et al., 2008; Honey et al., 2012). A top-down predictive strategy has also been suggested to facilitate chunking: learned lexical information facilitates the chunking of the new incoming input. This anticipatory strategy tries to predict future input to allow for a more effective recoding of the information when it is eventually sensed (Christiansen and Chater, 2016).

Decoding social calls requires mechanisms to keep track of temporal landmarks: onsets and offsets. The majority of cells in the auditory system are responsive to sound onsets, and cells with excitatory responses to offsets have been widely described (Qin et al., 2007; Gao and Wehr, 2015). While the linguistic role of sound onsets is clear, the function of explicitly encoding temporal offsets has been more difficult to deduce. One accepted function is gap detection in continuous sounds. In particular, between-channel gaps (interruptions between distinct events) have been implicated in the discrimination of voice onsets in consonants (Kopp-Scheinpflug et al., 2018). Distinct auditory pathways carry onset and offset information subcortically, but become integrated into A1 (Gao and Wehr, 2015).

Finally, social communication does not exist without context. Coarticulation is a microscopic example of context-dependency. In coarticulation, speech sounds influence the articulation of another in an anticipatory or carryover manner (Menzerath and de Lacerda, 1933; Ohala, 1993). At a macroscopic level listeners can predict upcoming words, based on the context, and incorporate them in ongoing linguistic processing (Van Berkum et al., 2005). Many context-dependent phenomena are due to syntactic regularities, which constrain the order of speech sounds. These regularities are exploited by the brain to reduce prediction errors and facilitate recognition (for potential mechanisms see section below; Leonard et al., 2015).

Animal studies have revealed potential network-level mechanisms underlying context dependency. An example is a sensitivity to rising or falling frequency sweeps, which is considered an important intonation cue in social communication. One candidate mechanism is the asymmetric organization of excitatory and inhibitory receptive fields in A1. There are populations of neurons with sideband inhibition that systematically changes its frequency bias along the tonotopic axis. This imparts directional selectivity (in frequency space) to neuronal responses (Zhang et al., 2003). Social communication circuits are also very sensitive to the fine temporal sequence of sounds. In a study of the rat A1, it was reported that neural networks could store estimates of tone order sequences for tens of seconds (Yaron et al., 2012). Nevertheless, neural mechanisms of this long-lasting temporal sensitivity remain unresolved (but see section below).

## Memory Demands for Grouping and Binding Speech Constituents

**Figure 2:**
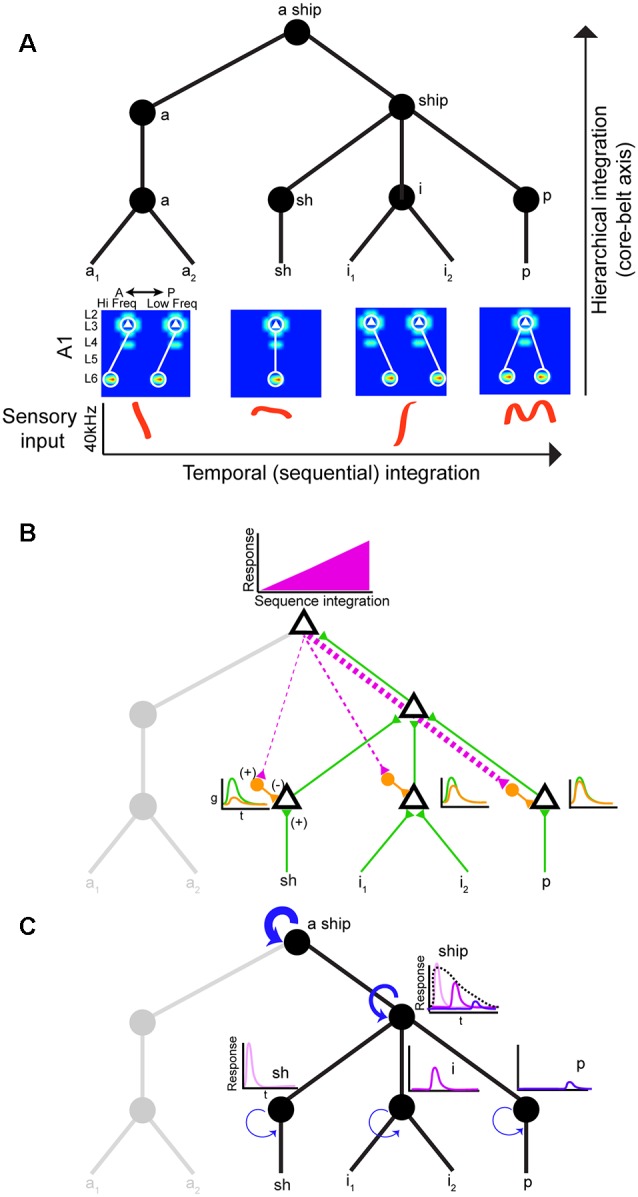
Hierarchical and sequential model of speech processing. **(A)** Phoneme detectors in A1 relay information to downstream areas that begin to form holistic representations. **(B)** Model of how predictive coding would alter the balance of excitation and inhibition in lower auditory areas. As the brain becomes more certain of upcoming sequences, feedback connections decrease prediction error by activating local inhibitory neurons in lower auditory areas. **(C)** Increasing temporal intergration windows could be achieved out by stronger recurrent actitvity in areas higher in the processing hierarchy.

Neural circuits that decode social signals need a way to group sounds into meaningful categories in a sequential manner. Hierarchical and sequential processing are widely accepted models of linguistic representation (Uddén et al., 2019). There is psychophysical and empirical evidence for hierarchical processing in humans (Levelti, 1970; Pallier et al., 2011). For instance, there are areas for processing language that are sensitive to increases in syntactic complexity: they show a progressive increase in activation as the number of components with syntactic meaning increases. This parametric change in activation suggests the processing of smaller-sized constituents (Pallier et al., 2011). The neural architecture underlying hierarchical processing of social calls is not known, but the graph theory framework suggests nested tree structures where hierarchical and sequential distances can be clearly distinguished (Figure 2A; Uddén et al., 2019). The assumption is that areas higher in the hierarchy can represent longer sequences due to longer windows of temporal integration. Therefore, mechanisms that support echoic memory are required for accumulating and holding information online (Furl et al., 2011). The activity of areas higher in the hierarchy would increase during sequence integration leading to increasingly stronger top-down input to areas lower in the hierarchy (Figure 2B). One potential mechanism to achieve predictive coding is inhibitory modulation from top-down input. These projections to lower areas can target local inhibitory neurons, which in turn reduce ongoing excitatory activity (Figure 2B; Pi et al., 2013). The prediction is that this increasing change in the balance of inhibition (top-down priors) and excitation (bottom-up) would suppress prediction error signals (Friston and Kiebel, 2009). In rodents, there is evidence of hierarchical auditory processing: responses to vocalizations become more invariant between A1 and the suprarhinal auditory field (Carruthers et al., 2015). It is worth noting that information flow in the auditory stream is more nuanced: it is both parallel and hierarchical with extensive cross-talk between each auditory subfield (Hackett, 2011).

Echoic memory is necessary to concatenate phonemes, syllables, words, sentences, so we can make sense of language holistically in real-time. Social calls are dynamic and fleeting requiring mechanisms to maintain memory banks. There is evidence that A1 can support sequence memories on the order of tens of seconds (Yaron et al., 2012), but no circuit mechanism has been directly implicated. One way to implement longer temporal integration windows is recurrent connectivity (Figure 2C; Wang et al., 2018). However, it remains to be shown how increasing temporal integration windows are being implemented in a hierarchical fashion between different areas. Studies on the computational demands of working memory can offer some mechanistic insight into how echoic memory can be implemented by neural circuits. These studies have proposed neural networks with temporal dynamics that provide stable firing past the stimulus presentation (i.e., persistent activity). In addition to strong recurrent connectivity, several neural mechanisms have been identified that can lead to stable persistent activity. One is the activation of synapses with NMDA receptors. The long-time constant of these glutamatergic receptors can sustain stable persistent activity. Also, some form of negative feedback to maintain control of the firing rate in the presence of strong recurrent connections would be required (Wang, 1999). It would be fruitful to examine whether these circuit features change systematically along the auditory cortical hierarchy.

## Circuit Foundation of Parallel Processing Between the Hemispheres

Lateralization is a widespread strategy in the nervous system to assign distinct computational tasks to the left and right hemispheres. For processing social communication, it is believed that the left and right A1 specialize in processing information at different temporal scales. The left A1 is postulated to specialize in fast syntactic processes (identifying specific sequences in speech), and the right in slower temporal information (prosody and intonation; Arnal et al., 2015). Lateralized language processing in humans has been known for over a century (Broca, 1861; Long et al., 2016) and is crucial for normal function (Oertel et al., 2010; Cardinale et al., 2013). This division of labor has also been observed in other species including rodents (Ehret, 1987; Marlin et al., 2015), and in a recent study we began to elucidate the circuit mechanisms that could underlie lateralization by comparing the connectivity of the left and right A1 (Levy et al., 2019). We found significant hemispheric differences in the connectivity of L3 pyramidal cells in the mouse A1. In the left A1 projections from L6 to L3 arose out-of-column from higher frequencies throughout most of the tonotopic axis. In contrast, the connectivity of the same pathway in the right A1 changed systematically with tonotopy, from lower to mixed to higher frequency bias. These distinct wiring schemes along the tonotopic axis suggest differences in spectrotemporal integration between the hemispheres.

To investigate the possible functional implications, we compared the responses to frequency sweeps of L3 neurons *in vivo*. In the left A1 there is a trend for L3 excitatory neurons to prefer downward sweeps regardless of their best frequency selectivity. This prevalent downward sweep selectivity could facilitate the left A1’s responsiveness to ethologically relevant sequences such as downward pitch jumps, which are common components of mouse vocalizations (Holy and Guo, 2005). Pitch jumps spanning several octaves would activate a subset of these asymmetric integrators, and binding of their individual responses would occur at an auditory region downstream (Figure 1). On the other hand, in L3 of the right A1 we found that sweep direction selectivity changed along the tonotopic axis and was inversely related to best frequency tuning: cells with high-frequency selectivity preferred downward sweeps, whereas cells with low-frequency selectivity preferred upward sweeps and in between cells had mixed selectivity. Similar trends have been observed in the rat’s right A1 (Zhang et al., 2003).

How does the brain dynamically control parallel, lateralized processes and information transfer without functional interference? Auditory sensory input stimulates both hemispheres and there are interhemispheric connections between the Auditory Cortices *via* the corpus callosum. One of the most debated questions is whether the impact of callosal projections is excitatory or inhibitory, which can determine the rules of information transfer and potential cooperativity. To a large extent the answer lies in the identity of the neurons targeted by callosal projections, but the targets and impact of interhemispheric projections on excitability remain unclear. A functional study in commissureless and normal primates suggested that the left A1 suppresses the right A1 (Poremba, 2006). Whereas hemispheric deactivation in cats suggested excitation is symmetric (Carrasco et al., 2013). In the rodent, almost every cortical layer (3, 4 and 5) is commissurally connected in a largely homotopic manner (Oviedo et al., 2010). Moreover, excitatory and inhibitory neuronal populations are commissural targets (Xiong et al., 2012). Hence, to answer the question of interhemispheric cooperation will require animal studies with very precise inactivation of specific neuronal classes in each hemisphere during vocal communication behavioral tasks.

## Conclusions and Future Directions

Human and animal studies should inform one another to gain a mechanistic understanding of how the brain decodes social communication. As recording techniques from humans improve, animal studies could serve as a guide to predict where (along the processing stream) and which signatures of specific auditory decoding operations should be examined. For instance, animal studies could help to unravel proposed neural mechanisms of stream segregation (Lu et al., 2017). Moreover, experiments in animals may also be established as a complementary invasive platform to study mechanisms hypothesized from human observational work. Animal models can serve as a comparative template to determine the relative contribution of sensory experience and genetic programs in the development of social-communicative functions. Communication deficits are the most common disabilities in children, affecting 8–12% of preschoolers. The underlying pathological mechanism routinely involves the miswiring of the connections between neurons in the language centers of the brain. Animal models can help identify critical time points, neural elements, and molecular targets to enable effective therapeutic interventions in human communication disorders.

## Author Contributions

HVO conceived and wrote most of the review article. DN helped write and revise the review article.

## Conflict of Interest

The authors declare that the research was conducted in the absence of any commercial or financial relationships that could be construed as a potential conflict of interest.
